# Suggestions for interprofessional educational courses from a students' perspective – a qualitative study

**DOI:** 10.3205/zma001212

**Published:** 2019-02-15

**Authors:** Veronika Schwarzbeck, Jan Hundertmark, Katja Wipfler, Cornelia Mahler, Susanne Frankenhauser, Jobst-Hendrik Schultz

**Affiliations:** 1 University Hospital Heidelberg, Department of General Practice and Health Services Research, Heidelberg, Germany; 2 University Hospital Heidelberg, Clinic for General Internal Medicine and Psychosomatics, Heidelberg, Germany; 3 University Hospital Heidelberg, Clinic for Anesthesiology, Heidelberg, Germany; 4 Eberhard-Karls University Tübingen, Department of Nursing Sciences, Tübingen, Germany; 5 BG Trauma Centre Ludwigshafen, Centre for Interdisciplinary Emergency Medicine, Ludwigshafen, Germany

**Keywords:** education, interprofessional relations, interdisciplinary communication, students, health occupations, suggestion

## Abstract

**Purpose:** The significance of interprofessional education (IPE) for interprofessional collaboration (IPC) and thus optimal patient care is widely recognised. As a starting point for a needs-based IPE development, we conducted focus groups interviews with students of different health professions. We assessed experiences with IPE and IPC, ascertained resulting IPE needs, and discussed opportunities for curricular implementation, to meet long-term challenges to health care.

**Methods: **Using semi-structured interview guides, we conducted five focus groups with altogether 18 trainees of different health professions and students of medicine and Interprofessional Health Care. We transcribed and analysed the data using qualitative content analysis.

**Results:** Three main categories of IPE approaches emerged out of the analysis: “content”, “settings”, and “challenges”. Contents as suggested by focus group participants are sessions about basic knowledge, practical skills, communication and interprofessional collaboration. The settings should comprise different forms and methods of teaching and didactic designs. As challenges, participants named unfamiliarity, teacher demands, differences in needs, diverging curriculum structures, heterogeneous prior knowledge, and aspects of timing.

**Conclusion: **Based on their prior experiences with IPC and IPE in other contexts, participants generated diverse ideas for new interprofessional courses and potential application in other IPC/IPE settings. This allows to integrate new IPE approaches into curriculum development.

## Zusammenfassung

**Zielsetzung:** Die Bedeutsamkeit interprofessioneller Lehre (IPE) für die interprofessionelle Zusammenarbeit (IPC) und eine damit optimierte Patientenversorgung ist bekannt. Zur bedarfsgerechten Entwicklung von IPE wurden Fokusgruppen mit Lernenden durchgeführt, die deren Erfahrungen mit IPE und IPC eruieren, resultierende Bedarfe an IPE ermitteln und Möglichkeiten zur curricularen Umsetzung diskutieren, um den heutigen Anforderungen an die Gesundheitsversorgung langfristig begegnen zu können.

**Methodik:** Anhand halbstandardisierter Leitfragebögen wurden fünf interprofessionelle Fokusgruppen mit insgesamt 18 Auszubildenden verschiedener Gesundheitsberufe, sowie Studierenden der Humanmedizin und der Interprofessionellen Gesundheitsversorgung durchgeführt und inhaltsanalytisch ausgewertet.

**Ergebnisse: **Im Bereich der „curricularen Ansatzpunkte“ ergaben sich die Hauptkategorien „Inhalte“, „Setting“ und „Herausforderungen“. Vorgeschlagene „Inhalte“ von den Lernenden sind Veranstaltungen zu Grundlagenwissen, praktischen Skills, Kommunikation und interprofessioneller Zusammenarbeit. Das „Setting“ soll verschiedene Unterrichtsformen und didaktische Besonderheiten enthalten. Als „Herausforderungen“ wurden Fremdheit, Anforderungen an die Lehrenden, unterschiedliche Bedürfnisse, unterschiedliche Strukturen in den Curricula und unterschiedliches Vorwissen, sowie zeitliche Aspekte benannt.

**Schlussfolgerung:** Die Teilnehmer konnten auf Basis ihrer bisherigen Erfahrungen mit IPC/ IPE vielfältige Ideen zu weiteren interprofessionellen Lehrveranstaltungen entwickeln. Diese entstanden aus in anderen Kontexten gewonnenen Erfahrungen, welche die Lernenden auf mögliche Anwendungen in IPE und IPC übertrugen. Die Curriculumsentwicklung hinsichtlich IPE soll nun auf Basis der gewonnen Daten weiterentwickelt werden.

## 1. Introduction

Interprofessional education (IPE) takes place, when “two or more professions learn about, from and with each other to enable effective collaboration and improve health outcomes” ([16], S. 13). Already in 2016, the WHO called for IPE to improve interprofessional collaboration (IPC) to master current challenges in health care, such as limited resources, increasingly complex health matters, as well as patient safety and complication rates [16]. The positive aspects of IPE are well established: In a review regarding the efficacy of interprofessional education, Reeves et al. identified improved capacities for teamwork as well as positive attitudes and increased knowledge about IPC as IPE outcomes [14]. Participation in IPE courses leads to positive changes in perception and attitudes concerning IPE/IPC [3], [15]; furthermore, participants recognised the importance of interprofessional teamwork [10]. Although IPE-related attitudes of students are well documented, relevant databases (PubMed, Cinahl) reveal little about students’ needs or proposals regarding IPE courses. In a needs analysis of German students, Konietzko et al. found a wish for further IPE and an interprofessional learning centre [9]. In a US study by Fitzsimmons et al., students of different health professions collaborated in the development of an integrated interprofessional teaching concept [2]. These students report a wish to work on authentic patient cases and learn in small-group exercises about differences in roles and responsibilities. Already in the (originally monoprofessional) frequently applied Kern-cycle of curriculum development [5], the students’ perspective is a relevant aspect of the cycle’s second step, needs assessment. Kern’s needs assessment entails the identification of both the intended and the current target group and environment characteristics. It aims to determine the desired competencies and the therefor necessary curricular design steps. The involvement of students, for instance regarding their current level of knowledge, their attitudes towards the current curriculum, or general strengths and weaknesses, is inevitable.

### 1.1. Objectives and study goals

Since 2012, interprofessional courses in communication, patient safety, business administration, and tutor trainings for students of medicine and Interprofessional Health Care B.Sc. (IPH) have been taking place at the Medical Faculty of Heidelberg University [1], [4], [7], [8]. Within the medical curriculum, these courses are usually offered as electives, but in the long run, we wish to provide and implement interprofessional education for all students. Fitzsimmons et al. give a first overview on typical student needs [2], but further, specific ideas by the affected learners themselves should be included. The goal of this study is to assess student perspectives on IPE and their ideas for potential interprofessional courses. This will contribute to IPE research and the expansion of the interprofessional programme at Heidelberg Medical Faculty.

## 2. Methods

To assess learners’ wishes and needs as well as their perspective on current challenges of IPE, we chose a qualitative approach using interprofessional focus groups (FG). FG are a type of group interview, in which the participating interviewees discuss predetermined questions about a specific given topic. In alignment with Kern’s recommendations, we as an interprofessional team developed a semi-structured interview guideline, which took the learners‘ needs and their relevant prior experiences into account [5] (see table 1 (Tab. 1)).

### 2.1. Participants and data collection

As suitable participants for the focus group interviews, we identified trainees at the Academy of Health Professions, students of IPH, and medical students who had already participated in one or more interprofessional courses or had previously completed a qualification in any health profession. This inclusion criterion within medical students was to ensure a sufficient familiarity with interprofessionalism in theory or practice. All potential participants received an information letter including study information. The focus group interviews took 50 to 70 minutes. All participants received a financial compensation of €15 for their time.

We aimed for and conducted five interprofessional focus group interviews with three or four interviewees to achieve a sufficient contentual saturation. Altogether 18 persons volunteered to participate: nine medical students, five IPH students (out of which two were trainees in a health profession, in addition to their studies), four trainees at the Academy for Health Professions. Age ranged between 19 and 56 years (M=25.2). The completed or ongoing professional trainings some participants had were nursing (N=7), physiotherapy (N=2), occupationaltherapy (N=1), medical laboratory assistance (N=1), orthoptics (N=1), medical radiography assistance (N=1), and speech and language therapy (N=1). The FG were conducted by an interprofessional team with members from the Department of General Practice and Health Services Research, the Clinic for Anesthesiology, and the Clinic for General Internal Medicine and Psychosomatics, from the Heidelberg University Hospital. Every session was audio and video recorded and held by one interviewer (VS, JH) and a minute taker.

#### 2.2. Data analysis

All FG were transcribed verbatim by means of the f4transkript software. The video footage served for correctly identifying the respective speakers during transcription. We analysed the data following the qualitative content analysis of Mayring [11], which allows systematic data interpretation with defined analysis rules and steps. The text material is sequentially reduced to inductive categories by generalising phrases and passage to more abstract key terms [11]. Three team members (VS, JH, KW) independently analysed the data and subsequently compared and discussed the found codes and categories until finding accordance. 

## 3. Results

In the qualitative content analysis, we identified six domains altogether: “definition of interprofessionalism”, “IPE experiences”, “IPC experiences”, “IPC-independent experiences”, “desired content and experiences regarding IPC”, and “curricular starting points”. This article focusses on “curricular starting points”, i.e. learners’ concrete ideas and suggestions for further IPE in their respective curricula. Our analysis yielded three main categories (“content”, “setting”, and “challenges”) with altogether 12 associated subcategories (see table 2 (Tab. 2)).

### 3.1. Main category: Content

This category encompasses concrete suggestions for future interprofessional courses from the learners’ point of view. The subcategory “basic knowledge” captures statements about different professions’ common contents that can potentially be learned in joint courses.

“I believe that depends on the content, well there are certainly topics that maybe make sense in a lecture-style, so that everyone in on the same page, but afterwards everyday practice should be in the foreground.” (FG3TN02)

In the subcategory “practical skills” learners describe skills that can potentially be taught together, such as working together on a clinical case (hereinafter the different sub-subcategories within the respective subcategories are highlighted in italics) or emergency exercises. Hereby, a shared language as a foundation for future cooperation can be developed, communication can be improved and what is important for every profession can be understood.

“Exactly, that is, how can I communicate in an emergency situation in a way that the other one understands and that I know what I’m expected to do…” (FG3TN01). 

Realistic/practical exercises, in which actual collaboration takes place, such as swallowing and drinking trainings, patient mobilisation or positioning, movement, or changing dressings, should be taught together.

“…these practical examples are kind of, to get a feel for it; it would make sense to, well, do that together, and not that the medical student acts as physician and as a nurse…” (FG3TN03).

The subcategory “communication” encompasses ideas concerning improved communication between professions, with patients, or their relatives.

“… that joint courses, for example with regard to communication, patient communication, or with family members could be offered.” (FG1TN01)

Learning a common technical language can strengthen mutual understanding. As to general patient interaction, breaking bad news should be practiced by all profession as well as collaborative medical briefings, interviewing techniques, or reflecting one’s own communication styles. Joint exercises in taking a patient’s history, potentially supported by simulation patient actors, can create further connections between professions. Within the subcategory “interprofessional collaboration”, we summarised the interviewees’ wishes to learn about and get to know other professions and overcome stereotypes. Work shadowing was mentioned as one possible opportunity to have a look at and feel into other professions’ fields of activity. 

“Well, first of course an explanation would be helpful: For instance, what is it that a physiotherapist or therapist actually does or what are the areas of responsibility in nursery. So that you first learn something about it in theory.” (FG2TN01)

Interfaces should be identified to optimise both learning from one another and future collaboration, as well as learning to identify and evaluate one’s own competences.

#### 3.2. Main category: Setting

Our analysis yielded “setting” as the second main category. The subcategory “forms of teaching” comprises statements about instructional designs suitable for IPE. This includes one-time events with opportunities of getting to know each other and come together in conversation, as well as joint courses. Traditional lectures are not helpful, as they offer no space for mutual exchange, although they may serve as an introduction to create a common ground for subsequent small-group exercises.

“… it’s no use saying: ok, I attend a lecture about whatever is slightly important for every subject, with different people, if it’s just chalk-and-talk teaching about interprofessionalism. I mean, I’m not interprofessional just because I listened to a two-hour talk about it, but instead I believe that, like in problem-based learning, we need to work in small groups.” (FG5TN04).

All courses, lectures, or events should take place on a voluntary basis, but offer incentives for participants. Occasionally, the interviewees voice a wish for reciprocial visits in their courses. Peer tutorials are one type of interprofessional learning setting, but peer tutors should be specifically trained in IPE. Moreover, the leadership role that medical students would potentially take as peer tutors was discussed critically, as it might solidify hierarchies on one hand, but on the other hand also provide an opportunity for forming collegial relationships.

As to e-learning, the interviewees see potential organisational barriers to equal access as well as little opportunity to benefit from IPE. However, e-learning might support individual preparation, create a common ground of knowledge, and help to organise learning groups or face-to-face encounters. Informal learning should generally be promoted so that learners may get to know each other and exchange ideas in a relaxed atmosphere. As potential obstacles to informal learning, interviewees named the physical distance between sites, different course hours, and general inhibitions to approach one another without a formal frame. In the “Didactic features” subcategory, participants wished for a change of roles in interprofessional courses.

“So that one knows how it feels – to be in the other‘s position, perhaps, for instance by reading some instruction in advance, like in a classical role-play. Well, obviously in that case, one would not act perfectly or even directly know what to do, but experience the other position, a change of perspective.” (FG3TN01).

Additionally, learners should work together on cases to gain insight into other fields of activity.

#### 3.3. Main category: Challenges 

The “challenges” main category encompasses all statements concerning potential difficulties in the implementation, organisation and realisation of IPE. In the “unfamiliarity” subcategory, participants name inhibitions to approach persons from other subjects, but rather stick to one‘s own group.

“It‘s obvious that if people know each other, they’ll rather talk to those they already know.” (FG4TN03)

The subcategory “differences in needs and demands” summarises difficulties due to differences in the respective necessary knowledge gains.

*“And I believe, well, the curricula need to overlap, and then the intensity and depth of learning might differ.” (FG1TN02).*

Further subcategories of challenges are demands towards “teachers”, “differences in prior knowledge”, “different structures”, as well as “time aspects” regarding the organisation of teaching units within the different curricula.

“…that would need to be coordinated, so that the nursing school informs about their courses and at some point the medical curriculum team organises their schedule accordingly, but that’s quite an effort to communicate. But I believe once that’s properly implemented, the whole procedure will be the same… and once that’s properly organised, it will be relatively simple, I believe, to adapt that from year to year.” (FG4TN01).

## 4. Discussion

The results show the large range of ideas and suggestions that learners have for IPE. They name both shared subject-specific knowledge and overarching content important for IPC. The study participants wished for more opportunities to get to know other professions as well as their respective fields of practice and establish a common language. As to teaching settings, participants prefer joint teaching units. Studying together in lectures may establish a common ground, but getting to know other professions and experiencing IPC should best take place in small groups. Participants further wish for teaching settings that stimulate new perspectives, empathising, and mutual understanding of members of other professions and their specific backgrounds, for instance by means of role-plays. Potential challenges are time constraints due to diverse curricula, differences in prior knowledge, and different needs and demands regarding course content.

In accordance with Konietzko et al. [9], the interviewees wished for more IPE and for learning content relevant to IPC. They were sensitive to potential challenges and, based on their prior experiences, came up with various ideas what and how should be learned in IPE. Gunaldo et al. [3] and Lumague et al. [10] similarly concluded that IPE is important for getting to know important aspects of IPC [3] and for the development of competencies crucial to IPC [10]. The interviewees further discussed whether IPE courses should teach specific, practice-oriented contents or overarching themes. Much like Fitzsimmons et al. [2], they wished for incentives for IPE course participation as well as small group learning settings to get to know the different health professions with their respective fields of activity, roles, and responsibilities. E-learning was viewed ambivalently, as study participants questioned the use of learning alone in front of a computer for promoting IPE. In their scoping review, Reeves et al. [13] reported a similar tendency towards isolation through e-learning and associated adverse effects on the courses‘ efficacy. Then again, however, multiple studies found that the advantages of e-learning, such as easy access and overcoming geographical borders, promoted IPC [13].

Apparently, even without experience in IPE implementation, the study participants were able to anticipate many challenges found in Nock‘s implementation expectations [12]. In 2016, Nock described an increase in organisational and temporal challenges associated with larger numbers of professions involved, as well as challenges in preparing teachers and dealing with different learning cultures [12].

Altogether, the interviewees had manifold ideas about contents and structure of interprofessional courses. Nevertheless, their suggestions were mainly inspired by prior experiences from other courses. Entirely new ideas were hardly presented, either because the already experienced methodologies provided sufficient clues for further IPE developments, or because the interview setting and guideline did not encourage the generation of further ideas.

This study‘s strengths are the interprofessional setting in the research teams and focus groups as well as the direct and creatives exchanges of ideas within the focus groups.

### 4.1. Limitations

Qualitative research does not aim for generalisations, but for assessing a spectrum of opinions to get a thematic overview. Therefore, the ideas regarding an expansion of interprofessional courses generated in this study do not necessarily represent the wishes of the majority of students. Therefore, one limitation is our approach‘s subjectivity, which does not offer objective criteria for the design of IPE courses. Moreover, the interprofessional focus groups potentially were subject to social desirability and therefore rather promoted consensus instead of primarily assessing the diversity of participants‘ opinions. Furthermore, participants may have mixed impressions from interprofessional and monoprofessional courses when discussing prior experiences with IPE.

## 5. Conclusions

Students are able to develop manifold ideas for interprofessional courses based on prior experiences with IPE and IPC and are therefore an important source of information. Their diverse suggestions, as gathered in interprofessional focus group interviews, generate ideas for IPE settings and anticipate typical implementation challenges. The students are very interested in the development of interprofessional courses, recognise the importance of IPC, and use focus groups to exchange ideas about opportunities to implement these courses. As a next step, based on this study, we will further develop our interprofessional curriculum. Future research should focus on students’ opinions on concrete course plans to gain deeper insight into this perspective on IPE. Moreover, expert interviews could advance these insights and may be less subject to social desirability than focus groups interviews.

## Ethics

This study was approved by the ethics committee of the Medical Faculty of Ruprecht-Karls University Heidelberg (number S-380/2016) and the University Hospital’s staff council.

## Acknowledgement

This study was supported by the project Sonderlinie Medizin by the State Ministry of Baden-Wuerttemberg for Sciences, Research and Arts. Our sincere thanks go to Lydia Oeljeklaus for interview transcription, Johanna Hoffmann for supporting data collection, and all focus group participants.

## Competing interests

The authors declare that they have no competing interests. 

## Figures and Tables

**Table 1:**
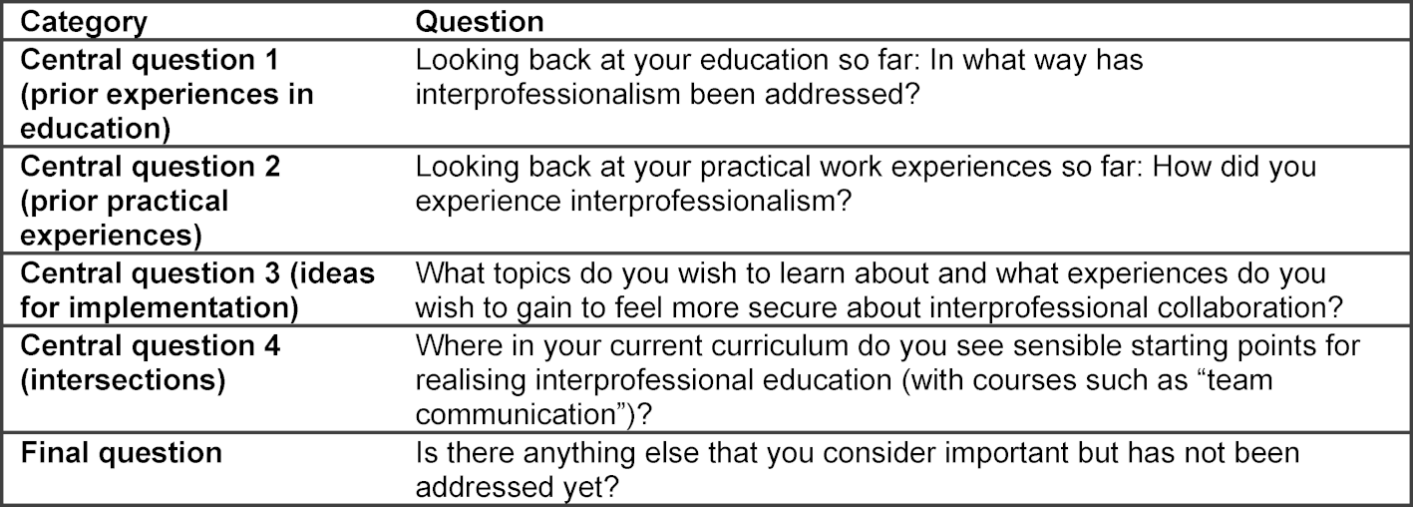
Central questions of the focus groups interview guideline.

**Table 2:**
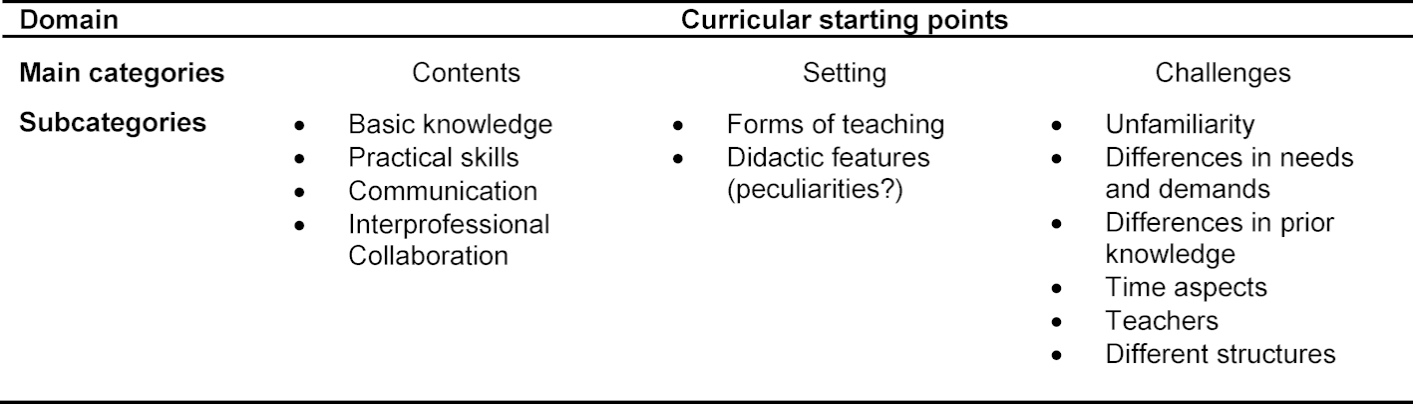
Main and subcategories within the curricular starting points domain
